# Entertainment or Health? Exploring the Internet Usage Patterns of the Urban Poor: A Secondary Analysis of a Randomized Controlled Trial

**DOI:** 10.2196/jmir.4375

**Published:** 2016-03-03

**Authors:** Rachel F McCloud, Cassandra A Okechukwu, Glorian Sorensen, Kasisomayajula Viswanath

**Affiliations:** ^1^ Dana-Farber Cancer Institute Center for Community-Based Research Boston, MA United States; ^2^ Harvard TH Chan School of Public Health Department of Social and Behavioral Sciences Boston, MA United States

**Keywords:** health communication, socioeconomic factors, information-seeking behavior, Internet

## Abstract

**Background:**

Important gaps remain in our knowledge of how individuals from low socioeconomic position (SEP) use the Internet for resources and in understanding the full range of activities they perform online. Although self-report data indicate that low SEP individuals use the Internet less than high SEP people for health information and for other beneficial capital-enhancing activities, these results may not provide an accurate overall view of online use.

**Objective:**

The aim of this study was to determine the ways in which low SEP individuals use the Internet, including for entertainment, social networking, and capital-enhancing functions, and how they are associated with health information seeking.

**Methods:**

Detailed Web tracking data were collected from 118 low SEP individuals who participated in the intervention group of a randomized controlled trial that provided Internet access. Websites were grouped by topic, including categories of capital-enhancing websites that provided access to resources and information. Different types of online activities were summed into an Internet use index. Single and multiple negative binomial regression models were fitted with the Internet use index as the predictor and health information seeking as the outcome. Next, models were fitted with low, medium, and high Web usage in capital-enhancing, entertainment, and social network categories to determine their associations with health information seeking.

**Results:**

Participants used the Web for diverse purposes, with 63.6% (75/118) accessing the Internet for all defined types of Internet use. Each additional category of Internet use was associated with 2.12 times the rate of health information seeking (95% CI 1.84-2.44, *P*<.001). Higher use of each type of capital-enhancing information was associated with higher rates of health information seeking, with high uses of government (incident rate ratio [IRR] 8.90, 95% CI 4.82-16.42, *P*<.001) and news (IRR 11.36, 95% CI 6.21-20.79, *P*<.001) websites associated with the highest rates of health information seeking compared to their lowest use categories. High entertainment website use (IRR 3.91, 95% CI 2.07-7.37, *P*<.001) and high social network use (IRR 2.06, 95% CI 1.08-3.92, *P*=.03) were also associated with higher health information seeking.

**Conclusions:**

These data clearly show that familiarity and skills in using the Internet enhance the capacity to use it for diverse purposes, including health and to increase capital, and that Internet usage for specific activities is not a zero sum game. Using it for one type of topic, such as entertainment, does not detract from using it for other purposes. Findings may inform ways to engage low SEP groups with Internet resources.

## Introduction

The vast quantities of online information have transcended some barriers to information, such as time and geography, to provide people with relevant, timely information that may increase their health and well-being [1]. The extent to which individuals are able to take advantage of available information and use the Internet to acquire benefits and opportunities [2-3] depends on their ability to navigate the online environment and requires diverse forms of online engagement [4-5]. Those who are unable to fully engage with these technologies are at a disadvantage to reap benefits that the online world may offer [1]. Digital inequalities describe the differing levels of ability to engage with the Internet among those with access [6], with research often emphasizing the differences in how individuals from lower socioeconomic position (SEP) incorporate the Internet into their lives compared to those with higher SEP [7-10]. However, focusing on a deficits approach in which low SEP individuals are compared to those with greater means may overshadow the myriad ways low SEP individuals benefit from the Internet. This study, from a randomized controlled trial involving low SEP individuals, used innovative, real-time Web tracking data to contextualize more fully the Internet information seeking of low SEP individuals.

Health information seeking may occur most often when need for a specific disease or medical decision making arises [1,11-14], but may not be indicative of everyday Internet use. Indeed, health information seeking serves as an important marker of disparities, with widespread evidence indicating that individuals from low SEP seek online health information less than their high SEP counterparts [11,15-17]. However, this seeking behavior must be placed in context of the entire spectrum of Internet engagement to fully understand the impact of digital inequalities.

Explorations of the ways low SEP individuals use the Internet, including the range and breadth of activities performed on the Internet for a variety of functions [3], may more fully contextualize the online environment for underserved groups. Beyond accessing health information, there is a need to participate in Internet-mediated economic, political, and social networks to take full advantage of what the online world has to offer and to meaningfully participate in an increasingly digital society [2,5,18]. “Capital-enhancing” (p. 606 [19]) information seeking represents exploring websites and topics that may enhance one’s life chances and offer upward mobility, such as helping with career advancement or consulting financial services, and serve to improve one’s life circumstances by increasing access to material and informational resources [20]. The inclusion of a range of topics that may impact health and well-being reflects a social determinants perspective, in which access to resources such as wealth, education, and adequate housing may impact health outcomes [21-22]. Specifically, capital-enhancing Internet use may be defined in this context as using the Internet to increase access to tangible and information-based resources that can address structural-level factors such as housing, education, employment, finances, and access to government resources.

Despite the potential benefits of such capital-enhancing information, research indicates that individuals with lower SEP take fewer opportunities to use the Internet comprehensively beyond amusement and communication, suggesting that the underserved may not fully take part in the new media environment [3]. Studies have found that, compared to individuals with higher income, low SEP individuals are less likely to use the Internet for capital-enhancing activities [19,23,24], instead using the Internet predominantly for entertainment purposes [20,23,25,26]. Such a “usage gap” between information and entertainment uses of the Internet may serve as an indicator of disparities, identifying who is not able to fully participate in and reap the benefits of the online world [18,19,23,26].

However, many studies reporting such a usage gap rely on self-report Internet use information [3,18,20,23,24], which is subject to the limitations of self-report data, such as bias and inaccuracy, particularly over longer periods of recall [27]. Relying on dichotomies of use (eg, never/ever use) of selected website categories from self-reported data may overgeneralize findings and may not accurately reflect or contextualize Internet usage in low SEP groups [3].

Additionally, providing a broad comparison between the Internet usage patterns of different income strata do not account for the differences in how low SEP individuals may engage with and learn from the Web in unique ways from their high SEP counterparts. Beyond the scope of entertainment versus information, scholars have suggested that a broader and more sophisticated use of the Internet, particularly engaging with the Web for diverse purposes, allows an individual a greater opportunity to acquire benefits and opportunities to meet individual and social goals [3]. The range and breadth of activities performed on the Internet, such as visiting a number of different types of sites that perform a variety of functions [3], may indicate that an individual is better situated to participate in a society that has transitioned many services online [4]. Use of the Internet for functions such as shopping, social network use, blogs, email, and reference sites are rarely captured in Internet studies and are even rarer if not nonexistent for low SEP individuals. Including an assessment of these activities from observed (tracked) Internet activity may provide useful context to Internet activities of interest, such as health information seeking, and if other types of Internet use serve to complement or substitute health seeking. Furthermore, they may indicate that the individual is more adept at using the Internet for a number of diverse functions.

Determining the detailed usage patterns of low SEP individuals may highlight the best ways to engage them in online activities that provide them with resources to improve their health or socioeconomic position.

The purpose of this paper is to build on our prior work [28] using a unique dataset containing directly tracked Internet use from Click to Connect (C2C), a randomized controlled trial that provided first-time at-home Internet access to the urban poor to explore their Internet behaviors. We were able to directly capture the Internet sites that participants visited to provide a detailed look at the Internet seeking behaviors of low SEP individuals. The nature of this study allowed us to capture all instances of information seeking in a natural setting. Previously, we examined the Internet browsing patterns of low SEP individuals and discovered that although Internet portals and social networking sites were the most used, the heterogeneity of the websites visited suggested the participants were using the Internet for a diverse range of functions [28]. In this study, we sought to provide a detailed description of the diverse ways that low SEP individuals use the Internet, including the range of functions individuals perform online, information seeking for specific beneficial functions, and entertainment use. Then, we explored how this use was associated with health information seeking within the context of everyday, home-based computer use in this low SEP sample.

## Methods

Data for this study were drawn from “Click to Connect: Improving Health Literacy Through Computer Literacy” (C2C), a randomized controlled trial funded through the National Cancer Institute to understand computer- and Internet-related challenges, barriers, and facilitators among a low SEP population. Intervention details may be found elsewhere (see [28,29]). Recruitment efforts were designed to recruit low-income, urban poor who are seldom represented in national surveys [29]. For the purposes of this study, we included data from the intervention group participants of all three waves of implementation who were given computers and Internet access during the course of the trial. Human participant approval for this study was granted by the Institutional Review Board (IRB) for the Dana-Farber Cancer Institute.

### Data Sources

Two sources of data were merged for this study: (1) a baseline 45-minute telephone survey that contained detailed measures of demographic information and (2) Internet use throughout the intervention period (9-18 months) tracked directly through participants’ computers using Spector 360, software that logs each URL visited into a secure server on the study premises through a virtual private network (VPN). The use of the Spector 360 process allowed us to capture real-time data of websites visited and number of times visited. Once all tracking data were collected, we submitted deidentified domain information to an online application program interface (API) through the Webroot BrightCloud Content Classification Service [30], which categorized each domain into one of 82 predetermined topic categories using a proprietary algorithm. These categories served as the basis for our Internet-based measures. Categorized websites then underwent additional crosschecks for accuracy for inclusion to the final list of topics used in our analysis. For example, we first reviewed the URLs for each categorized website to determine appropriateness for inclusion because websites encode useful words into the URL [31]. URLs that could not be immediately classified were entered into the search bar of Google for a brief website description. When needed, BrightCloud categories were combined to create larger groups of websites that better fit our definition of the topic. All survey and browsing data were merged for each study participant and analyzed in STATA version 13 (StataCorp LP, College Station, TX, USA). For reasons of confidentiality per the IRB mandate, we tracked household browsing activity rather than individual browsing information. Self-reported Internet use data from the survey were crosschecked with the browsing data to determine that there were no discrepancies in observed and self-reported Internet activity and that the participant was an active user of the Internet during the study.

### Measures

Internet health information seeking was conceptualized as the purposeful seeking of health information through visiting health websites. Our definition of “health” was broad to include all topics that participants may perceive as health information, including websites for health information of unknown quality, in order to capture health seeking from the participant’s perspective. Due to the broadened definition of health operationalized in this study, websites categorized by BrightCloud underwent a second, detailed coding process by study staff. We first created a list of health-related topics, derived from several sources, including the Healthy People 2020 topic list and Centers for Disease Control and Prevention and World Health Organization website indexes of health topics. We then used these keywords to search for additional URLs visited by the participants to add to the list of BrightCloud categorized health websites. Once a list of all potential health websites was created, two trained independent coders reviewed each URL and related website description and designated them as a health website (yes/no). A test coding block was first conducted with 10% of the sample to answer questions and clarify coding terms. Then, the two coders coded the full list independently and concurrently. The interrater reliability of the coding was strong, with a Cohen kappa of .94. The final list of health websites included sites such as the Cancer Society, , and HealthyPlace.com, among others. For the purposes of this study, each “hit,” or separate visit to a particular health-related website, was considered an instance of information seeking.

### Internet Use Index

We constructed an Internet use index corresponding to a number of different types of activities one may perform on the Web [3,18]. To construct this index, we analyzed Internet behavior for a diverse range of Internet functions, including social networks, streaming media, blogs, news, email, search engines, reference, Internet portals, and types of capital-enhancing seeking (described subsequently) as defined through our modified BrightCloud categories. The exact components of this index and related definitions of each can be found in Multimedia Appendix 1. To construct this index, each Web function was dichotomized as one (having visited at least one website in a particular category) or zero (meaning that the participant had not used the Web for this purpose). Dichotomized variables were then summed with higher scores equaling use of the Internet for a higher number of diverse purposes. The index included 16 potential categories for Internet use.

### Capital-Enhancing Information Seeking

Each category of capital-enhancing information seeking was coded as a separate variable and each hit was considered an instance of information seeking. Websites for each type of capital-enhancing seeking were derived from our modified BrightCloud categories. The category descriptions are described subsequently.

#### Education Information Seeking

Hits for information pertaining to higher education, including college websites, college-finding services, collegiate test preparation, GED courses or materials, and online degree program information.

#### Job Information Seeking

Hits for sites for information on employment, including human resources departments, job finders, or resume help.

#### Residence Information Seeking

Hits for information on renting, buying, or selling properties or real estate, including apartment listing services, roommate finders, and real estate websites.

#### Finance Information Seeking

Hits for money-related information, including banking services, loans, credit, accounting, stock trading, asset management, and investment accounts.

#### Government Information Seeking

Hits to websites for government agencies (local to national level), services, and explanation of laws, including political advocacy websites that promote politicians, political discussions, or other social advocacy issues.

#### News Information Seeking

Hits to websites for current events, including radio, newspaper and headline news sites, newswire services, personalized news, and weather sites.

### Entertainment Site Usage

Entertainment usage was derived from the modified BrightCloud categories and was also conceptualized as the number of hits to websites for sites discussing television, movies, music, celebrity news/gossip, entertainment reviews, or the performing arts. Sites for music, online gaming, nudity, and pornography were included. Examples of such websites included FreeGamesOnline.com, Access Hollywood, and IMDb.

### Social Network Site Usage

Social network usage was defined as the number of hits to sites that have user communities where users interact, post messages, pictures, and communicate, such as Myspace and Facebook.

### Covariates

We measured sex, race/ethnicity (white, African American, Latino), employment status (working yes/no), and age (categorized as younger than 35 years, 35-49 years, and 50 years or older) from our baseline telephone survey. Income and education were not included as covariates due to our recruitment of low SEP participants with a restricted income and education range. We also controlled for study wave to adjust for any differences by administration year.

### Analysis

We first analyzed descriptive statistics and frequencies for all variables. We next fitted an unadjusted and adjusted negative binomial regression model with our Internet use index and our outcome, Internet health information seeking. Negative binomial regression was used for these analyses due to the nature of the outcome as a count-based variable that had a strong right skew [32]. Potential confounders that were significant at the *P≤*.25 level with the predictor and outcome were entered into the adjusted analysis. We set this conservative alpha threshold to capture potential confounders that may not emerge as significant due to our small sample size. All capital-enhancing variables were first assessed as continuous variables to gain descriptive statistics on the range, median, and mean number of hits to each category. Then, each capital-enhancing category was divided by thirds into levels of low, medium, and high use based on number of hits, with low users as the referent group. They were then entered into unadjusted and adjusted negative binomial regression models with the outcome. We then combined all the hits from the capital-enhancing categories into one measure and divided it into three categories and followed the same process as described previously. We then repeated this process with entertainment and social network site usage as the predictor of interest.

## Results

The demographic characteristics of the sample can be found in Table 1, which illustrates the focus of C2C on low SEP groups compared to national communication surveys. The majority of the sample was black (55.1%, 65/118), with 80 of 118 (67.8%) older than 35 years, and 73 of 118 (61.8%) female. The sample was low income, with 33.1% (39/118) reporting a household income of less than US $10,000 per year and 90 of 118 (76.2%) reporting less than a high school degree.

**Table 1:** Demographic comparisons between Click to Connect (C2C) and selected national surveys.

Demographic characteristic		C2C, n (%) N=118	US Census 2010 N=308,745, 538^a^ [33]	HINTS 2014 Cycle 3, n (%) N=3185 [34]	Pew Internet Tracking Survey 2013, n (%) N=4178 [35]
**Sex**					
	Male	45 (38.2)	49%	1197 (37.58)	2059 (49.28)
	Female	73 (61.8)	51%	1906 (59.84)	2119 (50.72)
**Age (years)**					
	18-34	38 (32.2)	21% (20-34 years)	426 (13.38) (18-34 years)	926 (22.16) (<30 years)
	35-49	54 (45.8)	33% (35-59 years)	712 (22.35)	1329 (31.81) (30-49 years)
	50-64	26 (22.0)	12% (55-64 years)	1070 (33.59) (50-64 years)	1155 (27.64)
**Race/Ethnicity**					
	African American	65 (55.1)	13%	421 (13.22)	527 (12.61)
	White	8 (6.87)	78%	1584 (49.73)	3113 (74.51)
	Hispanic	23 (19.1)	16%	511 (16.04)	545 (13.04)
**Income (US$)**					
	<10,000	39 (33.1)	8%	680 (21.35) (<20K)	370 (8.86)
	10,000-19,999	37 (31.4)	6% (10K-<15K)		479 (11.46)
	20,000-29,999	19 (17.9)	11% (15K-<25K)	418 (13.12) (20K-<35K)	438 (10.48)
	30,000-39,999	9 (8.4)	10% (25K-<35K)		440 (10.53)
	40,000-49,999	3 (2.5)	14% (35K-<50K)	394 (12.37) (35K-<50K)	286 (6.85)
	50,000-74,999	2 (1.7)	18%	446 (14.00)	622 (14.89)
	≥75,000	0 (0)	32%	801 (25.15)	816 (19.53)
**Education**					
	≤Grade school	16 (13.6)	6%	297 (9.32) (≤high school)	312 (7.47) (<high school)
	Some high school	74 (62.7)	8%		
	High school graduate/ GED	3 (2.5)	50%	699 (21.94)	1401 (33.53)
	Some college	0 (0)	21%	691 (21.70)	1311 (31.38)
	≥Bachelor’s degree^b^	9 (7.6)	28%	1167 (36.64)	1143 (27.36)

^a^ Population estimate (exact numbers not available).

^b^ For C2C: college completed in another country.

### Description of Internet Information-Seeking Behaviors

The outcome, Internet health information seeking, received a median of 85.5 hits (range 0-3537; mean 214.59, SD 411.65 hits) over the study period (Table 2). The highest median number of hits for capital information–seeking variables was news information seeking, which had a median of 219 hits (range 0-8043) over the study period. Residence information seeking (median 9.5, range 0-2442 hits) was the lowest of all the categories. In comparison to the total universe of hits to all websites (Table 3), health information seeking represented 0.49% (24,972/5,084,901) of all hits over the study period. Social network sites received the highest number of hits of all categories.

**Table 2:** Descriptive statistics for online seeking for health, capital-enhancing variables, entertainment, and social networks by number of hits.

Type of seeking		Mean (SD)	Median (range)
Health		214.59 (411.65)	89 (0-3537)
**Capital-enhancing**			
	Job	234.53 (379.68)	61 (0-1832)
	Residence	119.03 (333.37)	10 (0-2442)
	Government	132.88 (204.65)	62 (0-1583)
	Education	175.81 (287.48)	70 (0-1470)
	Finances	505.20 (1052.30)	110 (0-7833)
	News	509.09 (993.48)	219 (0-8043)
Entertainment		4164.70 (6286.96)	1497 (0-31,023)
Social networks		15,740 (27,989.97)	4276 (0-169,875)

**Table 3:** Percentage of total hits contributed by each Web category.

Category		% of total hits n=5,084,901
Health information hits		0.49%
**Capital-enhancing hits**		5.97%
	Residence	0.29%
	Government	0.34%
	Education	0.41%
	Job	0.48%
	Financial	1.22%
	News	3.23%
Entertainment hits		9.74%
Social networks		36.54%
**Hits for other forms of Internet use**		
	Internet portals	12.63%
	Shopping	6.11%
	Search engine	5.64%
	Streaming media	5.01%
	Personal sites and blogs, peer-to-peer, shareware and freeware, personal storage	2.04%
	Society	1.81%
	Web-based email	1.74%
	Training and tools, reference and research, other education, translation	1.10%
Other websites visited (computer sites,^a^ malware, hacking, phishing, frauds, spyware, spam, dead sites)		10.62%

^a^ Web ads, Web hosting, parked domains, pay to surf, proxy, content and file delivery systems.

### Internet Use and Internet Health Information Seeking

All participants participated in at least 6 of 16 Web activities over the course of the study (Table 4). The majority (63.6%, 75/118) visited websites from all categories of use, and 18.6% (22/118) visited all but one category. Participants who engaged in greater Internet use had a significantly higher rate of health information seeking (Table 5); every additional Web category a participant visited was associated with an increase in the rate of health information seeking by a factor of 2.1 in the adjusted model (incident rate ratio [IRR] 2.12, 95% CI 1.84-2.44, *P*<.001).

**Table 4:** Percentage of participants using the Web for diverse purposes (N=118).

Number of website types visited	Participants, n (%)
7	3 (2.5)
8	2 (1.7)
11	1 (0.8)
12	3 (2.5)
13	6 (5.1)
14	6 (5.1)
15	22 (18.6)
16	75 (63.5)

### Capital-Enhancing Information Seeking and Health Information Seeking

As shown in Table 5, each type of capital-enhancing information seeking was significantly positively associated with Internet health information seeking and this relationship increased as usage increased. For example, participants who were medium users of education sites had 3.0 times the rate of Internet health information seeking compared to those who were low users of education sites when other factors were held constant (IRR 3.04, 95% CI 1.64-5.54, *P*<.001), whereas high users of education had 7.0 times the rate of health information seeking compared to low users (IRR 6.94, 95% CI 3.73-12.92, *P*<.001). Notably, high users of government sites had 9.0 times the rate of health information seeking compared to low users (IRR 8.90, 95% CI 4.82-16.42, *P*<.001) and high news site users had 11 times the rate of health information seeking compared to low users (IRR 11.36, 95% CI 6.21-20.79, *P*<.001) in adjusted models. When combined into a measure of total capital-enhancing Internet use, medium capital-enhancing site users had 4.2 times the rate of health information seeking compared to low capital-enhancing users (IRR 4.24, 95% CI 2.43-7.40, *P*<.001) when other factors were held constant and high capital-enhancing users had 13.1 times the rate of health information seeking of low users (IRR 13.01, 95% CI 7.29-23.20, *P*<.001).

### Entertainment Site Use and Health Information Seeking

Compared to low entertainment site users (Table 5), medium users sought health information at 3.3 times the rate when other factors were held constant (IRR 3.34, 95% CI 1.82-6.14, *P*<.001), whereas high entertainment site users sought information at 3.9 times the rate of low users (IRR 3.91, 95% CI 2.07-7.37, *P*<.001).

### Social Network Site Use and Health Information Seeking

High social network site users sought health information at 2.1 times the rate of low users in the adjusted model (IRR 2.06, 95% CI 1.08-3.92, *P*=.03).

**Table 5:** Bivariate and adjusted associations between each type of capital-enhancing seeking, entertainment site usage, social network site usage, and health information seeking (N=118).

Predictor variable			Bivariate associations		Adjusted models^a^	
			IRR (95% CI)	*P*	IRR (95% CI)	*P*
Multimodal use			2.16 (1.87-2.50)	<.001	2.12 (1.84-2.44)	<.001
**Capital-enhancing use**						
	**Financial site users (ref: low users)**					
		Medium	2.64 (1.42-4.90)	.002	1.93 (1.01-3.68)	.047
		High	5.19 (2.81-9.59)	<.001	5.13 (2.81-9.34)	<.001
	**Education site users (ref: low users)**					
		Medium	2.570 (1.41-4.68)	.002	3.04 (1.64-5.54)	<.001
		High	6.75 (3.72-12.23)	<.001	6.94 (3.73-12.92)	<.001
	**Residence site users (ref: low users)**					
		Medium	2.89 (1.51-5.41)	.001	2.16 (1.11-4.19)	.02
		High	3.96 (2.10-7.46)	<.001	3.91 (2.03-7.53)	<.001
	**Job search site users (ref: low users)**					
		Medium	3.23 (1.75-5.97)	<.001	3.05 (1.65-5.64)	<.001
		High	5.79 (3.13-10.69)	<.001	6.17 (3.28-11.62)	<.001
	**Government site users (ref: low users)**					
		Medium	4.14 (2.31-7.43)	<.001	4.82 (2.64-8.80)	<.001
		High	8.90 (4.98-15.91)	<.001	8.90 (4.82-16.42)	<.001
	**News site users (ref: low users)**					
		Medium	4.91 (2.77-8.71)	<.001	5.87 (3.32-10.38)	<.001
		High	11.29 (6.38-19.96)	<.001	11.36 (6.21-20.79)	<.001
	**Total capital site users (ref: low users)**					
		Medium	4.69 (2.72-8.09)	<.001	4.24 (2.43-7.40)	<.001
		High	14.77 (8.59-25.39)	<.001	13.01 (7.29-23.20)	<.001
**Entertainment site users (ref: low users)**						
	Medium		3.65 (1.97-6.76)	<.001	3.34 (1.82-6.14)	<.001
	High		4.66 (2.49-8.73)	<.001	3.91 (2.07-7.37)	<.001
**Social network site users (ref: low users)**						
	Medium		0.93 (0.47-1.85)	.85	1.04 (0.52-2.08)	.92
	High		2.15 (1.14-4.08)	.02	2.06 (1.08-3.92)	.03

^a^ Adjusted for race, age, native language, employment status, and wave.

## Discussion

This study represents in-depth research of natural online behaviors of low SEP individuals over a period of several months that draws from directly tracked Internet data. Through this method, we were able to place health information seeking, capital-enhancing information seeking, entertainment use, social network use, and other diverse forms of Internet use within the context of the total Web use experience of low SEP individuals, data that are not often captured in such detail for this group. Participants sought information on a number of domains; 64% visited all the categories of the Internet use index over the study period and each additional category of computer use was associated with double the rate of health information seeking. Higher use of all individual types of capital-enhancing seeking was associated with increased rates of Internet health information seeking, with the highest increases seen in education and governmental website use. When all capital-enhancing categories were combined, the highest users of capital information sought health information at 13 times the rate of low users. Furthermore, both medium and high entertainment users were significantly associated with higher health information seeking compared to those who used entertainment sources to a low extent, and high social network site use was associated with higher health information seeking compared to low use.

Broader use of the Internet may provide individuals with skills to become more active online consumers [3]. Indeed, once online, low SEP individuals in the current study used the Internet for a diverse range of functions, from information gathering to sharing data to using search engines to springboard to other types of information. Importantly, this increased use was associated with increases in health information seeking. Although this behavior may present differently from that of a high SEP seeker, it points to the diverse ways in which low SEP individuals use the Web to find numerous types of content, including health information. This also indicates that even those who have not had much prior Internet use experience are able to quickly acclimate to the Web and use it for diverse functions beyond just entertainment once they are provided with access.

Evidence shows that health outcomes are patterned by access to material resources, education, and occupation [22]. By engaging in capital-enhancing information seeking, participants may gain an increase in resources that improves their SEP and subsequently improves their health. Thus, capital-enhancing functions may be important to consider when understanding how low SEP groups are using the Internet. Although all capital categories were significantly associated with higher Internet health information seeking, the notable rates of seeking associated with news, government, and education uses of the Internet may highlight the importance of these functions working to complement one another for the goal of greater well-being. For example, the literature has documented how online governmental information seeking and using the Internet as a civic resource and forum strongly influences civic engagement [36]. It may be that those who are more likely to research current events, governmental and political information, and engage with their communities may be more prone to research information pertaining to other avenues to well-being; participants may also visit government sites to access services such as food stamps or housing assistance.

In all, these associations suggest that higher levels of Internet use for functions such as searching for a job, financial resources, or educational programs correspond with higher levels of searching for health information. Past literature has found that in certain contexts, financial information served as a competing concern to health information for low SEP individuals [37]. In contrast, this study supports a conceptualization of complementation of seeking for types of beneficial sites instead of substitution. In other words, instead of the search for money or adequate housing replacing health information seeking due to competing needs, different types of capital-enhancing information may work together to provide a more comprehensive set of resources to increase well-being.

Although participants visited entertainment websites more frequently than health or capital websites reflecting past literature, we also observed entertainment use of the Internet was positively associated with greater health information seeking. This is an important distinction; instead of entertainment use taking precedence over higher-order activities, individuals who spend more time online may do so in varied topic areas as they gain more confidence in using the Web [18] and as complex skills required for Web 2.0 uses develop [38]. This may also indicate that once individuals gain more exposure to the Internet, their perceived utility of the Web for different functions increases. Such findings are an important distinction, particularly in a media environment that often portrays low SEP individuals as using entertainment content to the extent that it overshadows other forms of Internet use. For example, a recent *New York Times* article claimed that increased access to technology creates a “time-wasting gap” in which the entertainment uses of the Internet by low SEP individuals eclipse its use for positive, educational uses, particularly for youth [39]. In contrast, our study illustrates that Internet behaviors must be placed in context of the larger seeking environment; although entertainment content was highly accessed, high entertainment users were also more likely to use the Internet to gather health information.

Although both health and capital information seeking represented only a small total of all websites visited compared to categories such as social networking, Internet portals, and entertainment, it may be indicative of the nature of the sites’ structure. For example, sites with constantly changing content, such as celebrity gossip, and particularly user-generated content, such as social network sites, may require more frequent interaction to remain up-to-date with activity. However, static sites may only require one visit to gather needed information, such as referencing a health diagnosis. Other sites, such as job sites, may be visited only as a certain need arises. The frequency with which these dynamic sites, such as social network sites, are accessed provide a promising platform for future eHealth content delivery [28]. This may point to more diverse use of social network sites for patient support and delivery of important health information, particularly because they are already a familiar platform for many Internet users. Social network sites also represent an important source of information exchange and a growing number of Internet users are turning to these sites to post about their own health activities, follow friends’ health experiences, or find health information [40].

### Limitations

Although these data may give us valuable insight into the information-seeking behaviors of lower income adults, this sample may not be generalizable to other low SEP Internet users in the Boston area or in the United States. Although the restricted range of our sample precluded us from gauging differences by income or education, the nature of the sample made it ideal for studying the communication behaviors of a low SEP group. For this study, we recruited participants through presentations at adult education centers, which may have led to selection bias. Our focus on adults aged 25 to 60 years precludes us from understanding Internet usage patterns in younger adults, who are often more frequent Internet users; however, this allowed us to focus on a sample of novice Internet users who may not have as much exposure to the Internet through school or other sources. Due to our IRB mandate, our data were restricted to the household level, so we were unable to determine use from particular individuals in the household. It is possible that there were several users in each household and that different household members used the Internet for different purposes. To address this discrepancy, self-report Internet use data from each participant was crosschecked with website tracking data to determine if the participant’s level of reported usage matched the observed level of Internet use in the tracking data. Furthermore, additional models accounting for other potential household members did not change our regression estimates. Our use of BrightCloud coding to determine our topic categories may have limited us from including certain relevant websites in our analyses; although we conducted a second-level crosscheck to include or exclude inappropriately categorized sites, we may have overlooked certain URLs or we may be unable to determine if health information seeking occurred on a site such as a social network platform or multipurpose webpage. Despite these limitations, the ability to capture real-time, Web-recorded data provides valuable insight into the information-seeking behaviors of the urban poor.

### Conclusion

Results indicate that once online, low SEP individuals use the Internet for a broad range of purposes. The growth of health information technologies provides opportunities to incorporate features of interactivity and multimedia to revolutionize health communication. As evidenced by the diverse Web behaviors in this group, they may be familiar with these concepts and well positioned to participate in upcoming Web interventions. This finding may have important implications for interventions and design of policy-based websites because low SEP individuals may take advantage of a number of different well-being and health-related website formats. Given the popularity of social network sites, this platform may be particularly suited for trusted, reliable health information. However, certain safeguards to information structure, accessibility, and content must be considered when designing Web resources for low SEP groups.

## Appendix

Multimedia Appendix 1 Definitions of website categories.

## Abbreviations

C2C: Click to Connect
IRB: Institutional Review Board
IRR: incident rate ratio
SEP: socioeconomic position
