# Supplementary material for: Entertainment or Health? Exploring the Internet Usage Patterns of the Urban Poor: A Secondary Analysis of a Randomized Controlled Trial
Source: J Med Internet Res. 2016 Mar 3;18(3):e46. doi: 10.2196/jmir.4375 (PMC4796406; doi:10.2196/jmir.4375)
Supplement: Multimedia Appendix 1 [file jmir_v18i3e46_app1.pdf]

Appendix 1: Definitions of Website Categories

Category	Definition [30]
Personal Sites and Blogs	Personal websites posted by individuals or groups, blogs.
Internet Portals	Web sites that aggregate a broader set of internet content and topics, and which typically serve as the starting point for an end user.
Search Engines	Search interfaces using key words or phrases. Returned results may include text, websites, images, videos.
Streaming Media	Sales, delivery, or streaming of audio or video content, including sites that provide downloads for such viewers.
Reference and Research	Personal, professional, or educational reference material, including online dictionaries, maps, census, almanacs, library catalogues, genealogy, and scientific information.
Email	Sites offering web based email and email clients.
Society	A variety of topics, groups, and associations relevant to the general populace, broad issues that impact a variety of people, including safety, children, societies, and philanthropic groups.
Shopping	Department stores, retail stores, company catalogs and other sites that allow online consumer or business shopping and the purchase of goods and services.
Social Networks	Social networking sites that have user communities where users interact, post messages, pictures, and communicate, such as MySpace and Facebook.
Education	Information pertaining to higher education, including college websites, college finding services, collegiate test preparation, GED courses or materials, and online degree program information.
Job	Information on employment, including human relations departments, job finders, or resume help.
Residence	Information on renting, buying, or selling properties or real estate. Includes apartment listing services, roommate finders, and real estate websites.
Finance	Banking services, loans, credit, accounting, stock trading, asset management, and investment accounts.
Governmental	Government agencies (local to national level), services, and explanation of laws. Also incudes political advocacy websites that promote politicians, political discussions, or other social advocacy issues.
News	Current events or contemporary issues. Also includes

	radio stations and magazines, newspapers online, headline news sites, newswire services, personalized news services, and weather sites.
Entertainment	Television, movies, music, celebrity news/gossip, entertainment reviews, or the performing arts. Includes sites for music, online gaming, nudity, pornography.
